# Association of serum Asprosin concentrations with heart failure

**DOI:** 10.1186/s12872-023-03668-z

**Published:** 2023-12-14

**Authors:** Guoan Wang, Chunzhen Fan, Yaru Chai, Xin Yu, Mingqing Xing, Zhihua Lv, Shanshan Yuan, Hongyan Dai

**Affiliations:** 1 School of Medicine and Pharmacy, Ocean University of China, Qingdao, Shandong China; 2 Department of Cardiology, Qingdao Municipal Hospital, Qingdao, Shandong China; 3 Department of Critical Care Medicine, The Second Affiliated Hospital of Air Force Medical University, Xi’an, Shaanxi China; 4 Department of Clinical Laboratory, Qingdao Municipal Hospital, Qingdao, Shandong China

**Keywords:** Asprosin, Novel adipokines, Heart failure

## Abstract

**Background:**

To analyze the association of serum Asprosin concentrations with heart failure (HF).

**Methods:**

A total of 103 patients with HF were included in the HF group, and 103 patients with health checkups were included in the non-HF group. The serum Asprosin levels of the two groups were measured, and relevant clinical data were collected for statistical analysis.

**Results:**

Compared with the non-HF group, the serum Asprosin concentration was significantly higher in the HF group, and the difference was statistically significant (*P* < 0.001). According to the serum Asprosin levels, we divided all the subjects into three quartiles. We found that the prevalence of HF increased with increasing serum Asprosin levels in the three groups (*P* < 0.001). Serum Asprosin levels were positively correlated with NT-ProBNP (*P* < 0.05) and negatively correlated with LVEF (*P* < 0.001). Dichotomous logistic regression analysis found Asprosin and age to be independent risk factors for HF (OR = 1.010, 95% CI: 1.003–1.018; OR = 1.058, 95% CI:1.004–1.665, respectively). Combining Asprosin and NT-proBNP indicators to draw ROC curves can improve the specificity and sensitivity of HF diagnosis.

**Conclusions:**

Serum Asprosin levels were significantly elevated in HF patients. The serum Asprosin level is an independent risk factor for HF, and the combined detection of Asprosin and NT-proBNP levels can improve the accuracy of HF diagnosis.

## Background

Heart failure (HF) is the end stage of many cardiovascular diseases, and its prevalence and mortality rate remain high, with approximately 15 million HF patients in 51 countries represented by the European Society of Cardiology [1]. Approximately 13.7 million people aged ≥ 35 years in China suffer from HF [2]. Despite the encouraging progress in drug and device therapy for HF in recent years, the mortality rate and readmission rate of HF patients in the first three months after discharge from the hospital reach 15% and 30%, respectively, and the 5-year mortality rate of chronic HF patients is more than 50%. Therefore, it is essential to investigate the factors affecting and predicting HF and explore new therapeutic targets.

Asprosin, a novel adipocytokine discovered in 2016 by Romere et al. [3], is synthesized and released by white adipose tissue mainly during fasting and is associated with appetite, glucose metabolism, insulin resistance (IR), and apoptosis. Asprosin has been found to play an important and complex role in metabolic and metabolic diseases, with significantly increased serum Asprosin levels in patients with obesity [4], type 2 diabetes mellitus (T2DM) [5], diabetic nephropathy (DN) [6], polycystic ovary syndrome (PCOS) [7], and nonalcoholic fatty liver disease (NAFLD) [8]. In addition, recent studies have found an important role in cardiovascular disease, with studies showing that serum Asprosin levels are significantly increased in patients with coronary artery disease (CAD) [9]. High serum asprosin levels combined with the number of coronary artery stenoses can diagnose the severity of CAD [10]. In vitro studies have confirmed that Asprosin upregulates ATP-binding cassette (ABC) transporters A1 (ABCA1) and ABCG1 expression through activation of the p38/Elk-1 signaling pathway, inhibits lipid accumulation in macrophages, and reduces the atherosclerotic burden in apoE-/- mice [11]. Whereas fewer studies have been conducted on the relationship between Asprosin and HF, only one study confirmed that circulating Asprosin levels were significantly higher in patients with dilated cardiomyopathy than in healthy controls and that patients with lower plasma Asprosin levels had a higher risk of adverse cardiovascular events than patients with higher plasma Asprosin levels [12]. Dilated cardiomyopathy is a rare cause of HF, and the results of this study cannot be directly applied to patients with other causes of HF. Therefore, the aim of this study was to investigate the correlation between serum Asprosin levels and HF with common clinical causes, with the aim of providing new ideas for the early diagnosis and management of HF.

## Methods

### Study subjects

From August 2021 to August 2022, 103 patients with HF who attended Qingdao Municipal Hospital in accordance with the Chinese Heart Failure Diagnosis and Treatment Guidelines 2018 were included in the HF group, including 64 males and 39 females with a mean age of 70.39 ± 10.66 years. Among them, 61 cases (59.2%) had ischemic cardiomyopathy, 39 cases (37.9%) had hypertensive heart disease, and 3 cases (2.9%) had rheumatic valvular heart disease. In the same period, 103 people without HF were used as the non-HF group, including 49 males and 54 females with a mean age of 68.17 ± 5.58 years. Patients with acute coronary syndrome, cardiogenic shock, obstructive pulmonary disease, severe hepatic and renal insufficiency, malignancy, mental disorders, and inability to cooperate were excluded. All study subjects signed an informed consent form, and the study was approved by the Ethics Committee of Qingdao Municipal Hospital.

### General clinical data and various biochemical indicators

The participants’ sex, age, body mass index (BMI), systolic blood pressure (SBP), diastolic blood pressure (DBP), previous medical history (hypertension, diabetes, and CAD), and applied drugs (ARB/ACEI/ARNI, β-blockers, calcium antagonists, diuretics, nitrates, dagliflozin, antiplatelet agents) were recorded. Venous blood from all participants was collected for the measurements of N-terminal pro-brain natriuretic peptide (NT-proBNP), fasting blood glucose (FBG), glycosylated hemoglobin (HbA1C), liver and kidney function, and lipid levels on the following morning after 8 h of fasting or more. Echocardiography-related indexes were recorded: left ventricular ejection fraction (LVEF), interventricular septum thickness (IVS), left ventricular posterior wall thickness (LVPW), left atrial internal diameter (LAD), left ventricular end-diastolic internal diameter (LVEDD), E/A value, and E/e ′value.

### Serum asprosin assay

Blood samples were obtained in the morning after 8 h of overnight fasting and centrifuged at 1000×g for 20 min after standing for 2 h at room temperature in dry tubes without anticoagulant, and the supernatant was extracted and stored in a -80 °C refrigerator. The serum Asprosin level was determined by enzyme-linked immunosorbent assay (ELISA) in strict accordance with the kit instructions.

### Statistical methods

The obtained data were statistically analyzed in the SPSS 26.0 program. Statistical significance was accepted as *P* < 0.05 in all analyses. The fit of the data to the normal distribution was checked with the Kolmogorov‒Smirnov test. The findings were averaged (mean ± standard deviation) for each continuous parameter, and an independent samples t test was used to analyze parametric data. One-way ANOVA was used between multiple groups, and further two-way comparisons were made using the LSD-t test or the Gaimes-Howell test. Information not conforming to the normal distribution is denoted by M (P25, P75), whereas the Mann–Whitney U test was used to analyze significant differences in nonparametric data. Comparisons between multiple groups were made using the Kruskal‒Wallis rank sum test and a two-by-two comparison. Categorical values are presented as numbers (n) and percentages (%) in tables and graphs, and comparisons between groups were made using the chi-square test. Pearson’s correlation test was performed to determine the relationship between the variables. Further multiple linear regression analysis was performed, and a linear equation was fitted. Logistic regression was used to analyze the factors influencing HF. A receiver operating characteristic curve (ROC) was used to assess the diagnostic value of asprosin in patients with HF.

## Results

### Comparison of clinical data and various biochemical indexes between the HF and non-HF groups

There were no significant differences in age, sex, BMI, systolic blood pressure (SBP), diastolic blood pressure (DBP), alanine aminotransferase (ALT), urea nitrogen (BUN), or E/A between the two groups. Total cholesterol (TC), triglycerides (TG), low-density lipoprotein cholesterol (LDL-C), aspartate aminotransferase (AST), creatinine (Cr), FBG, HbA1C, NT-ProBNP, IVS, LVPW, LAD, LVEDD and E/e were significantly higher in the HF group than in the non-HF group. The prevalence of hypertension (HBP), diabetes (DM), CAD, and the proportion of oral dapagliflozin, antiplatelet agents, statins, ACEI/ARB/ARNI, β-blockers, calcium blockers (CCB), diuretics, and nitrates were also significantly higher in the HF group than in the non-HF group, with statistically significant differences (*P* < 0.05). High-density lipoprotein cholesterol (HDL-C), apolipoprotein A1 (ApoA1) and apolipoprotein B (Apo B) were significantly lower in the HF group than in the non-HF group (*P* < 0.05) (Table 1).

**Table 1:** Comparison of clinical and laboratory parameters between the non-HF group and HF group

Variables	non-HF (N = 103)	HF (N = 103)	P
Gender (M/F)	49/54	64/39	0.069
Age (years)	68.17 ± 5.58	70.39 ± 10.66	0.063
BMI (kg/m^2^)	23.60 ± 3.52	24.35 ± 3.74	0.141
SBP (mmHg)	124.83 ± 18.17	127.83 ± 16.37	0.215
DBP (mmHg)	72.85 ± 14.35	74.8 ± 10.89	0.275
TC (mmol/L)	4.79 ± 1.00	3.69 ± 1.03^*^	0.000
TG (mmol/L)	1.26(0.96,1.90)	0.91(0.74,1.31)^*^	0.000
HDL-C (mmol/L)	1.18 ± 0.27	0.97 ± 0.25^*^	0.000
LDL-C (mmol/L)	2.79 ± 0.69	2.18 ± 0.74^*^	0.000
Apo A1 (g/L)	1.35 ± 0.20	1.08 ± 0.24^*^	0.000
Apo B (g/L)	0.97 ± 0.24	0.84 ± 0.34^*^	0.001
ALT (U/L)	14.74(11.93,22.3)	18.09(15.37,20.45)	0.717
AST (U/L)	14.81(10.23,27.00)	20.87(16.91,26.35)^*^	0.000
Cr (µmol/L)	66.42 ± 11.98	82.07 ± 18.17^*^	0.000
BUN (mmol/L)	6.58 ± 1.81	7.07 ± 2.32	0.069
FBG (mmol/L)	5.24 ± 0.98	6.24 ± 2.07^*^	0.000
HbA1C (%)	5.82 ± 1.36	6.57 ± 1.48^*^	0.000
NT-ProBNP (ng/L)	63.87(30,102.5)	1239.16(518,3535)^*^	0.000
IVS (mm)	8.54 ± 1.11	9.53 ± 1.58^*^	0.000
LVPW (mm)	8.46 ± 1.07	9.17 ± 1.1^*^	0.000
LAD (mm)	34.49 ± 3.87	44.48 ± 7.72^*^	0.000
LVEDD (mm)	45.21 ± 3.90	52.55 ± 8.63^*^	0.000
E/A	0.99 ± 0.34	1.15 ± 0.71	0.058
E/e′	8(7, 10)	13(11, 18)^*^	0.000
LVEF (%)	61.09 ± 2.51	43.5 ± 13.35^*^	0.000
Prevalence of DM	12(11.7%)	42(40.8%)^*^	0.036
Prevalence of HBP	35(34%)	83(80.6%)^*^	0.000
Prevalence of CAD	6(5.8%)	86(83.5%)^*^	0.000
Dapagliflozin	7(6.8%)	29(28.2%)^*^	0.000
Antiplatelet class	6(5.8%)	43(41.7%)^*^	0.000
Statins	28(27.2%)	82(79.6%)^*^	0.000
ACEI/ARB/ARNI	20(19.4%)	74(71.8%)^*^	0.000
β-blockers	13(12.6%)	82(79.6%)^*^	0.000
CCB	11(0.7%)	26(25.2%)^*^	0.006
Diuretics	2(1.9%)	93(90.3%)^*^	0.000
Nitrates	1(1%)	35(34%)^*^	0.000

BMI: mean body mass index; SBP: systolic blood pressure; DBP: diastolic blood pressure; TC: total cholesterol; TG: triglycerides; HDL-C: high-density lipoprotein cholesterol; LDL-C: low-density lipoprotein cholesterol; ApoA1: apolipoprotein A1; Apo B: apolipoprotein B; ALT: alanine aminotransferase; AST: aspartate aminotransferase; Cr: creatinine; BUN: urea nitrogen; FBG: fasting blood glucose; HbA1C: glycosylated hemoglobin; NT-proBNP: amino-terminal brain natriuretic peptide; IVS: interventricular septum; LVPW: left ventricular posterior wall; LAD: left atrial diameter; LVEDD: left ventricular end-diastolic internal diameter; LVEF: left ventricular ejection fraction; DM: diabetes; HBP: hypertension; CAD: coronary heart disease; CCB: Ca^2+^-blockers

*For comparison with the non-HF group, P < 0.05

### Comparison of serum asprosin concentrations between HF and non-HF groups

**Fig. 1:**
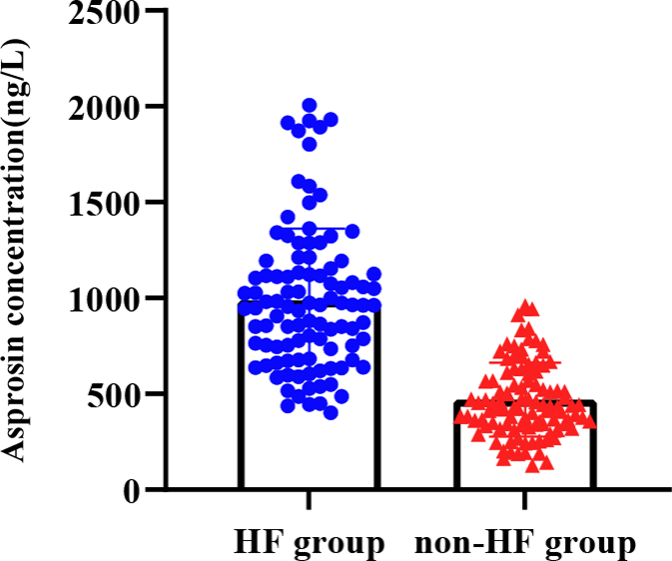
Comparison of serum Asprosin concentrations between the HF group and the non-HF group

The serum Asprosin level in the HF group (1030 ± 460 ng/L) was significantly higher than that in the non-HF group (470 ± 190 ng/L), and the difference was statistically significant (*P* < 0.001) (Fig. 1).

### Comparison of clinical data and biochemical indexes of serum asprosin trichotomies

**Fig. 2:**
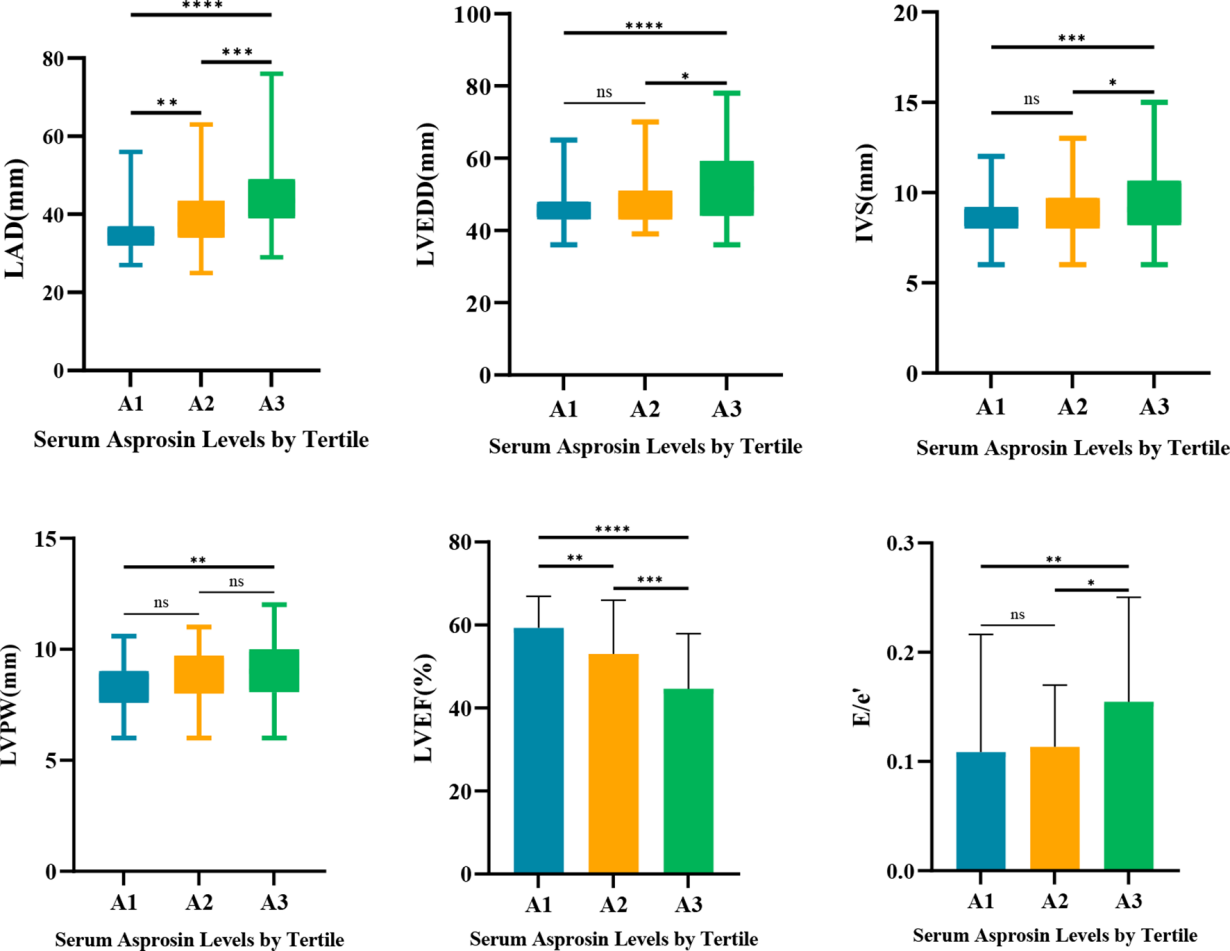
Comparison of echocardiography parameters by trisection grouping of serum Asprosin levels. IVS: Interventricular septum; LVPW: Left ventricular posterior wall; LAD: Left atrial diameter; LVEDD: left ventricular end-diastolic internal diameter; LVEF: left ventricular ejection fraction. *P < 0.05, **P < 0.01, ***P < 0.001

All subjects were divided into group A1 (Asprosin < 490 ng/L), group A2 (490 ng/L < Asprosin < 850 ng/L), and group A3 (Asprosin > 850 ng/L) according to serum Asprosin levels in tertile groups. There were significant differences in TC, TG, HDL-C, LDL-C, ApoA1, ApoB, AST, Cr, BUN, FBG, HbA1C, NT-ProBNP, IVS, LVPW, LAD, LVEDD, E/e′, LVEF, prevalence of DM, prevalence of HBP, prevalence of CAD and prevalence of HF among the three groups. In addition, ApoA1, Cr, NT-proBNP, the prevalence of HBP, CAD and HF, and LAD increased significantly with increasing serum Asprosin levels, while LVEF decreased with increasing Asprosin levels, and the differences were statistically significant (*P* < 0.05) (Table 2; Fig. 2).

**Table 2:** General clinical and laboratory parameters of all subjects according to serum Asprosin levels

Variables	A1 (N = 70)	A2 (N = 66)	A3 (N = 70)	P
BMI (kg/m^2^)	24.79 ± 3.75	23.99 ± 3.45	24.14 ± 4	0.411
SBP (mmHg)	125.56 ± 17.11	124.64 ± 17.20	128.69 ± 17.62	0.358
DBP (mmHg)	74.3 ± 11.62	73.8 ± 12.53	73.37 ± 14.12	0.912
TC (mmol/L)	4.72 ± 1.01	4.16 ± 1.15^*^	3.84 ± 1.14^*^	0.000
TG (mmol/L)	1.2(0.94,1.79)	1.02(0.73,1.41) ^*^	1.07(0.81,1.49) ^*^	0.010
HDL-C (mmol/L)	1.17 ± 0.28	1.07 ± 0.25	0.98 ± 0.28^*^	0.000
LDL-C (mmol/L)	2.67(2.42,3.07)	2.4(1.87,2.92) ^*^	2.26(1.61,2.8) ^*^	0.000
Apo A1 (g/L)	1.33 ± 0.21	1.23 ± 0.25^*^	1.08 ± 0.25^*#^	0.000
Apo B (g/L)	0.93(0.78,1.1)	0.83(0.69,1.03)	0.79(0.59,1.03) ^*^	0.016
ALT (U/L)	15.69(11.86,22.95)	14.73(10.79,21.82)	13.72(10.59,28.99) ^*#^	0.786
AST (U/L)	18.08(15.08,21.23)	18.83(16.04,22.02)	21.12(15.99,26.99) ^*#^	0.010
Cr (µmol/L)	66.77 ± 12.62	74.08 ± 16.72^*^	82.5 ± 18.68^*#^	0.000
BUN (mmol/L)	5.44(4.55,6.54)	5.87(4.94,7.28)	7.03(5.76,9.34) ^*#^	0.000
FBG (mmol/L)	5(4.64,5.89)	5.13(4.72,5.74)	5.54(5.01,7.08) ^*#^	0.003
HbA1C (%)	5.97 ± 1.58	5.93 ± 1.08	6.68 ± 1.56^*#^	0.004
NT-ProBNP (ng/L)	79(36.15,120.88)	113.91(51.91,889.66) ^*^	1328(521.18,4663.31) ^*#^	0.000
Prevalence of DM	10(14.3%)	15(22.7%)	29(41.4%)^*#^	0.001
Prevalence of HBP	27(38.6%)	38(57.6%)^*^	53(75.7%)^*#^	0.000
Prevalence of CAD	6(8.6%)	31(47%)^*^	55(78.6%)^*#^	0.000
Prevalence of HF	6(8.6%)	31(47%)^*^	66(94.3%)^*#^	0.000

BMI: Mean body mass index; SBP: systolic blood pressure; DBP: diastolic blood pressure; TC: Total cholesterol; TG: Triglycerides; HDL-C: High-density lipoprotein cholesterol; LDL-C: Low-density lipoprotein cholesterol; Apo A1: Apolipoprotein A1; Apo B: Apolipoprotein B; ALT: Alanine aminotransferase; AST: Aspartate aminotransferase; Cr: Creatinine; BUN: Urea nitrogen; FBG: Fasting blood glucose; HbA1C: Glycosylated hemoglobin; NT-proBNP: amino-terminal brain natriuretic peptide; DM: Diabetes; HBP: Hypertension; CAD: Coronary heart disease; HF: Heart failure

*indicates a significant difference compared to group A1

^#^indicates a significant difference compared to group A2

### Correlation between serum Asprosin levels and various factors

**Fig. 3:**
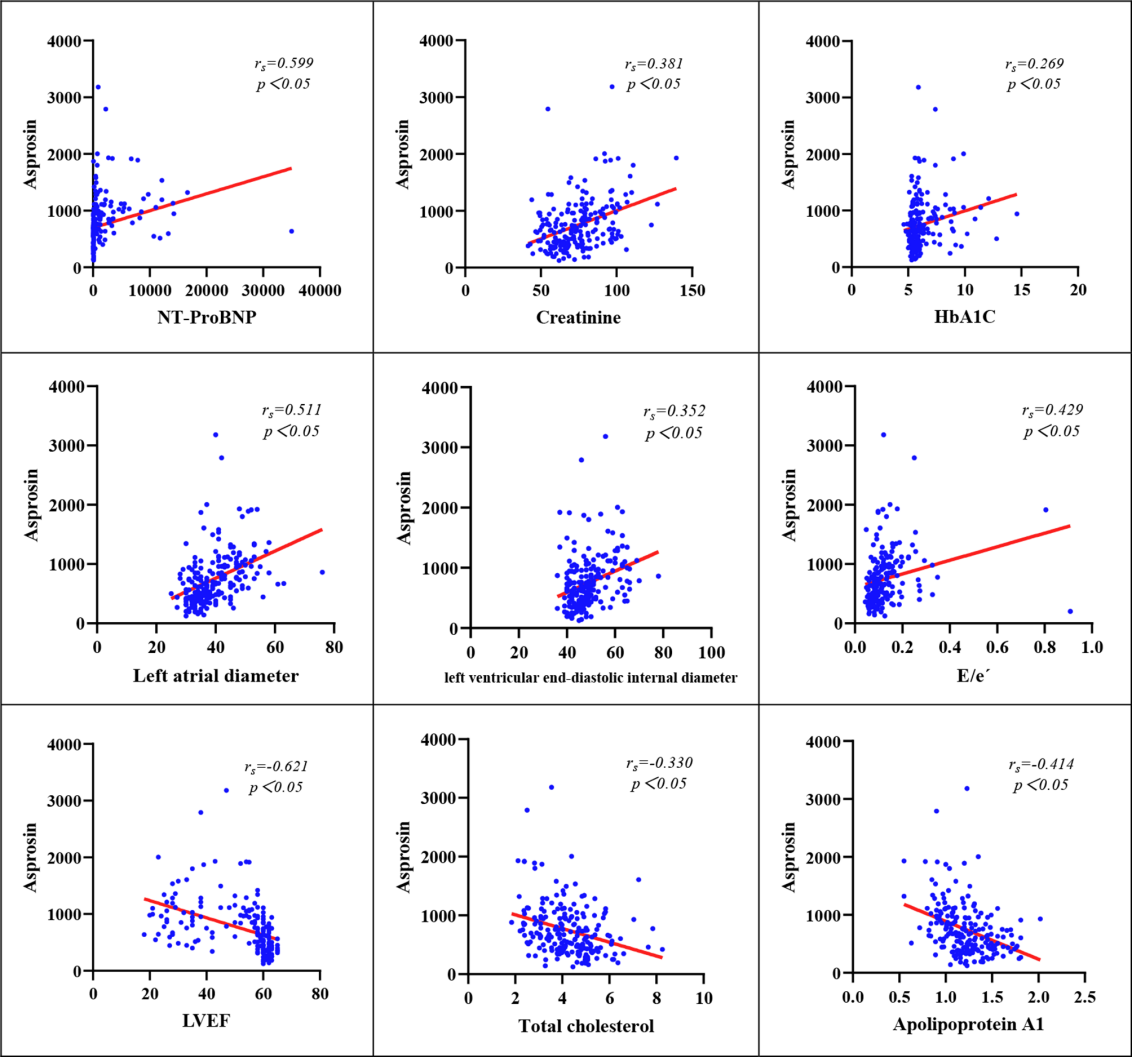
Correlation between serum Asprosin levels and other variables in the HF group

Spearman correlation analysis of the correlation between serum Asprosin levels and various factors revealed that serum Asprosin levels were positively correlated with NT-ProBNP, Cr, HbA1C, LAD, LVEDD, and E/e′ and negatively correlated with LVEF, TC, and ApoA1 levels (P < 0.05) (Fig. 3).

### Logistic regression analysis of factors influencing HF

Simple regression revealed that Asprosin, NT-ProBNP, TC, TG, HDL-c, LDL-c, ApoA1, Apo B, BUN, Cr, FBG, HbA1C, LVEF, IVS, LVPW, LAD, LVEDD, gender, age, no CAD and no DM were the influential HF factors, with statistically significant differences. Multivariate logistic regression analysis reported that serum Asprosin and age are HF-independent risk factors. However, LVEF and no CAD are independent protective factors for HF. (P < 0.05) (Table 3).

**Table 3:** Logistic regression analysis for the presence of HF

	Simple regression			Multiple regression	
	OR(95%CI)	P		OR(95%CI)	P
Asprosin (ng/L)	1.008(1.006–1.010)	<0.05		1.010(1.003–1.018)	<0.05
NT-ProBNP (ng/L)	1.015(1.008–1.032)	<0.05		-	-
TC (mmol/L)	0.327(0.229–0.467)	<0.05		-	-
TG (mmol/L)	0.446(0.283–0.703)	<0.05		-	-
HDL-c (mmol/L)	0.036(0.010–0.128)	<0.05		-	-
LDL-c (mmol/L)	0.289(0.181–0.460)	<0.05		-	-
Apo A1 (g/L)	0.003(0.000-0.017)	<0.05		-	-
Apo B (g/L)	0.188(0.065–0.541)	<0.05		-	-
BUN (mmol/L)	1.826(1.480–2.252)	<0.05		-	-
Cr (µmol/L)	1.071(1.047–1.096)	<0.05		-	-
FBG (mmol/L)	1.703(1.303–2.225)	<0.05		-	-
HbA1C (%)	1.569(1.203–2.046)	<0.05		-	-
LVEF (%)	0.432(0.325–0.575)	<0.05		0.614(0.439–0.860)	<0.05
IVS (mm)	1.753(1.379–2.230)	<0.05		-	-
LVPW (mm)	1.832(1.386–2.422)	<0.05		-	-
LAD (mm)	1.370(1.259–1.491)	<0.05		-	-
LVEDD (mm)	1.197(1.128–1.271)	<0.05		-	-
Gender (M/F)	1.808(1.038–3.150)	<0.05		-	-
Age (years)	1.118(1.084–1.152)	<0.05		1.058(1.004–1.665)	<0.05
No CAD	0.012(0.005–0.032)	<0.05		0.019(0.001–0.313)	<0.05
No DM	0.192(0.093–0.393)	<0.05		-	-

NT-proBNP: amino-terminal brain natriuretic peptide; TC: total cholesterol; TG: triglycerides; HDL-C: high-density lipoprotein cholesterol; LDL-C: low-density lipoprotein cholesterol; Apo A1: apolipoprotein A1; Apo B: apolipoprotein B; Cr: creatinine; FBG: fasting blood glucose; HbA1C: glycosylated hemoglobin; LVEF: left ventricular ejection fraction; IVS: interventricular septum; LVPW: left ventricular posterior wall; LAD: left atrial diameter; LVEDD: left ventricular end-diastolic internal diameter

### ROC curve analysis

**Fig. 4:**
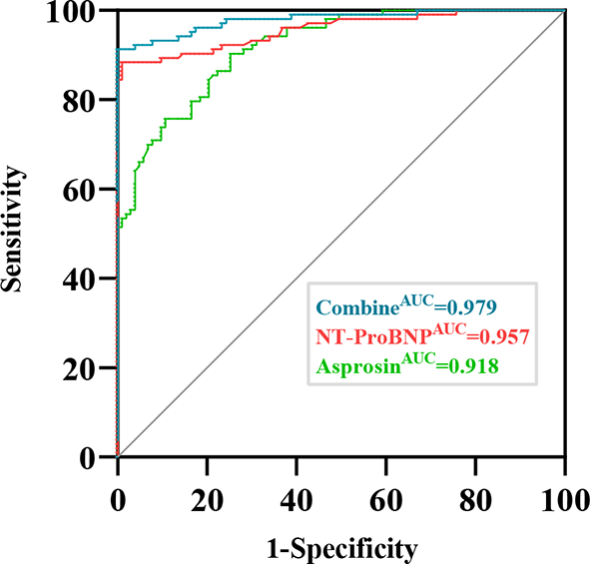
ROC analysis of Asprosin and NT-ProBNP in the diagnosis of HF

The ROC curve showed that the best cutoff point for serum Asprosin level to predict the occurrence of HF was 734 ng/L (sensitivity 75.7%, specificity 89.3%, Jorden index 0.65, area under the curve AUC 0.918). The area under the curve for NT-proBNP was 0.957, the sensitivity was 88.3%, the specificity was 99%, and the Jorden index was 0.874. Combining asprosin and NT-proBNP, the ROC curve was plotted with an area under the curve of 0.979 (95% CI: 0.962–0.997, *P* < 0.01), sensitivity of 91.3%, specificity of 99%, and Jorden index of 0.903 (Fig. 4).

## Discussion

The main finding of the current research is that circulating Asprosin concentrations were significantly increased in patients with HF. Furthermore, LVEF was an independent factor associated with Asprosin concentrations in HF. In addition, asprosin was independently and positively correlated with the occurrence of HF. Collectively, these findings suggest that Asprosin might be a risk factor related to the development of HF. To our knowledge, this is the first study to certify asprosin as a risk factor for HF.

Asprosin was identified as a novel hormone secreted by white adipose tissue (WAT) in 2016. It targets the liver, exerts a glucogenic effect [3], and leads to the impairment of insulin sensitivity and secretion. Emerging data suggest that it may exert various effects implicated in many cardio-metabolic diseases, including obesity [4], T2DM [5], PCOS [7], NAFLD [8], OSAS [13] and heart disease [10, 14]. Previous studies have reported elevated circulating Asprosin levels in adults with increased body weight [15]. Serum asprosin levels exhibited significant positive correlations with BMI, waist circumference, and glycemic, lipidemic, and proinflammatory parameters [16]. Notably, the existing data on circulating Asprosin levels in children with obesity are conflicting. Some studies reported increased levels [17], while others reported decreased levels [18]. Elevated levels of circulating Asprosin have been found in patients with T2DM compared to healthy controls [19], and circulating Asprosin levels may be related to the progression of kidney disease in patients with T2DM [20]. Circulating asprosin levels have also been found to be significantly higher in patients with PCOS [7], NAFLD [8], and OSAS [13].

Studies about asprosin and heart diseases or atherosclerosis are scarce and inconclusive. Serum Asprosin was found to be higher in CAD patients than in non-CAD subjects, and high levels were associated with an increased risk of developing CAD [9]. Asprosin values were found to be significantly higher in the multiple coronary lesions group than in the medical, single coronary, and double coronary lesion groups [10]. The level of serum Asprosin in the T2DM with carotid plaque group was significantly higher than that in the T2DM without carotid plaque group, and serum Asprosin was significantly correlated with carotid plaque in T2DM patients after adjusting for multiple confounding factors [21]. According to the mechanism studies, the results were paradoxical. Zou et al. reported that asprosin inhibits macrophage lipid accumulation, reduces atherosclerotic burden and demonstrates a protective role for asprosin in atherosclerosis development [11]. However, You et al. demonstrated that asprosin directly induces endothelial-to-mesenchymal transition and participates in vascular injury by activating the TGF-β signaling pathway [22].

Wen et al. [12] reported that patients with dilated cardiomyopathy had significantly higher circulating Asprosin levels than a healthy control group. Patients with lower plasma Asprosin levels had a greater risk of an adverse cardiovascular event than those with high plasma Asprosin levels. This study further demonstrated that the underlying protective mechanisms of Asprosin may be linked to its functions relating to enhanced mitochondrial respiration under hypoxia. Unlike Wen’s study, our research confirmed that Asprosin was a risk factor for the development of HF. In our research, circulating Asprosin concentrations were significantly increased in patients with HF and independently correlated with the occurrence of HF (OR is approximately 1.010). In addition, LVEF was negatively correlated with asprosin. The reasons underlying the different results in these two studies should be as follows: (1) These two studies recruited different types of HF patients. Wen’s study focused on HF patients with dilated cardiomyopathy, while our study recruited HF patients with different etiologies, including 61 (59.2%) with ischemic cardiomyopathy, 39 (37.9%) with hypertensive heart disease, and 3 (2.9%) with rheumatic valvular heart disease. (2) Previous studies have confirmed that high serum Asprosin levels are important risk factors for CAD [9] and T2DM [17] pathogenesis. Given that CAD and T2DM are the most critical risk factors for HF, we have reason to speculate that Asprosin can promote the occurrence and development of HF. Asprosin is a newly discovered adipokine; its research in the field of HF is very limited and controversial, so its exact role needs to be further elucidated.

In Wen’s study, they found that patients with lower or higher Asprosin levels exhibited similar serum BNP concentrations. However, in our research, NT-ProBNP levels increased in correspondence to tertiles of Asprosin, and serum concentrations of Asprosin were significantly correlated with NT-ProBNP. These results suggest that elevated BNP and asprosin in HF may have a common regulatory mechanism. ROC curve analysis shows that the combination of Asprosin and NT-proBNP has higher diagnostic efficiency, suggesting that Asprosin combined with BNP can be used as a more potential biomarker for the diagnosis of HF, providing a new reference for clinical diagnosis and treatment.

This study also has certain limitations. First, it is difficult to deduce the exact causal relationship between serum Asprosin levels and HF due to the cross-sectional study design. Hence, a larger sample of prospective studies is required to confirm this relationship. Second, this study focuses on the Chinese population; therefore, it needs to be carefully extended to other ethnic groups. Third, our research only detected serum levels based on ELISA, and there might have been some random measurement errors.

## Data Availability

The datasets generated and analyzed in this study are available from the corresponding author on request.

## Abbreviations

ABCA1 ATP: binding cassette transporters A1
ALT: Alanine aminotransferase
ApoA1: Apolipoprotein A1
Apo B: Apolipoprotein B
AST: Aspartate aminotransferase
BMI: Body mass index
BUN: Urea nitrogen
CAD: Coronary artery disease
CCB: Calcium blocker
Cr: Creatinine
DBP: Diastolic blood pressure
DM: Diabetes
DN: Diabetic nephropathy
ELISA: Enzyme-linked immunosorbent assay
FBG: Fasting blood glucose
HbA1C: Glycosylated hemoglobin
HBP: Hypertension
HDL-C: High-density lipoprotein cholesterol
HF: Heart failure
IVS: Interventricular septum thickness
LAD: Left atrial internal diameter
LDL-C: Low-density lipoprotein cholesterol
LVEDD: Left ventricular end-diastolic internal diameter
LVEF: Left ventricular ejection fraction
LVPW: Left ventricular posterior wall thickness
NAFLD: Nonalcoholic fatty liver disease
NT-proBNP: N-Terminal pro-brain natriuretic peptide
PCOS: Polycystic ovary syndrome
ROC: Receiver operating characteristic curve
SBP: Systolic blood pressure
T2DM: Type 2 diabetes mellitus
TC: Total cholesterol
TG: Triglycerides
WAT: White adipose tissue
